# A comparison of acute affective responses, physiological measures and training volume between superset and traditional resistance training in untrained adults

**DOI:** 10.3389/fspor.2025.1536747

**Published:** 2025-01-17

**Authors:** Per Aslak Myraunet, Atle Hole Saeterbakken, Vidar Andersen

**Affiliations:** Faculty of Education, Arts and Sports, Western Norway University of Applied Sciences, Sogndal, Norway

**Keywords:** strength training, time-efficient, RPE, RPD, sPDF, EES, feelings

## Abstract

The aim of this study was to compare the perceptive responses, physiological measures, training volume and training duration comparing a superset vs. a traditional resistance training session in untrained adults. Thirty adults (29 ± 7 years, 1.72 ± 0.1 m, 77 ± 16 kg) performed one superset resistance training session and one traditional resistance training session in a randomized-crossover design. Both sessions consisted of eight exercises with two sets and a load of ∼10-repetition maximum. The outcomes included number of repetitions, training duration, blood lactate and heart rate in addition to rate of perceived exertion (RPE), rate of perceived discomfort (RPD), session displeasure/pleasure (sPDF) and exercise enjoyment (EES) which were recorded in the middle and post-exercise. Forty-eight hours after the last session the participants reported which session they would prefer as their regular routine if they had to choose. The main findings were that the superset session led to greater RPE compared to the traditional session (*p* = 0.012–0.16, *d* = 0.53–0.54). Further, there was a trend towards greater RPD after the superset session, although not reaching statistical significance (*p* = 0.092, *d* = 0.41). There were no differences for sPDF (*p* = 0.404) or EES (*p* = 0.829). Furthermore, the superset session demonstrated higher levels of blood lactate levels (18.3%. *p* < 0.001, *d* = 0.81) and average heart rate (7.8%, *p* < 0.001, *d* = 1.53) compared to the traditional session. The traditional session took 60% longer time (*p* < 0.001, *d* = 6.62), and had 4.6% more repetitions (*p* = 0.006, *d* = 0.54) compared to the superset session. Two out of three participants reported the superset session as their preferred regular training routine. In conclusion, the superset session led to a higher perceived effort and discomfort, higher metabolic stress, took less time, had a lower training volume and was more preferred compared to the traditional session in untrained adults.

## 1 Introduction

Resistance training (RT) is associated with numerous positive health outcomes such as improved cardiovascular health and reduced risk of all-cause mortality (1, 2). Hence, performing RT two or more days per week for the major muscle groups has been recommended for adults by both the American College of Sport Medicine and the World Health Organization (3, 4). However, only one out of three adults fulfill the weekly RT recommendations (5, 6) where lack of time is one of the most commonly reported barriers to not perform RT (7, 8). Consequently, time-efficient RT strategies has been investigated and suggested as an option to increase the compliance to RT programs (9).

Superset, i.e., performing two exercises after the other with no, or limited, rest between them (10), has been proposed as a time-efficient method for RT, as it roughly halves the training time compared to traditional RT (11, 12). Superset has shown to induce greater neuromuscular fatigue (13) and increased levels of metabolites such as blood lactate, creatin kinase concentration and testosterone compared to traditional RT session (14–16). Further, the increased level of metabolic stress could potentially influence the total training volume in a RT session which may potentially have an effect on muscle strength and hypertrophy (17, 18). Nonetheless, studies comparing volume between superset and traditional RT shows conflicting results (13, 19–22) with some studies report no differences in volume (21, 22), while others report a significantly higher volume for superset compared to traditional RT (13, 20). Importantly, several of these studies investigated its effects across only two exercises (13, 20–22) and the results may not be transferable to RT sessions including several exercises muscle groups. To the best of our knowledge, only one study has compared volume between superset and traditional in a whole-body RT session (19). Andersen et al. (19) compared a traditional and supersets session consisting of 8 exercises with 3 sets per exercise at an intensity of 9-RM in resistance trained adults. The superset session led to a decrease in training volume (measured as completed repetitions) by 4.2% compared to the traditional session. These findings indicate the importance of examining complete training sessions, as that superset's limiting effects on volume first becomes apparent when training volume is substantial.

How physical activity is perceived has shown to influence an individual's choice to continue with that activity later (23, 24). If compliance to RT is of importance, this could be of equal important as time-efficiency as it is unlikely to continue with something that is not pleasant or enjoyable. To the authors best knowledge, the previously mentioned study is also the only study that has compared the affective responses between superset and traditional RT when conducting a full-body workout. Andersen et al. (19) demonstrated a significantly higher rating of perceived effort and discomfort in the superset session, and a tendency for the superset session to be more pleasurable compared to traditional RT. There was no difference for enjoyment between the protocols (19). Furthermore, 62% of participants reported that they would prefer to continue with the superset RT if they had to choose (19). Notably, the study is limited by not measuring any physiological measures, e.g., heart rate or blood lactate. Adding such measurements could provide additional insight to the relationship of physiological measures and affective responses to superset RT. Furthermore, Andersen et al. (19) included RT individuals, and the affective responses may not be representative for other populations e.g., untrained individuals. Finally, the study did not report the affective measures during the sessions, hence it is unknown how or if the affective responses changed during the sessions. Therefore, the primary aim of this study was to compare the affective responses during and at the end of a whole-body superset vs. a traditional RT session in untrained individuals. Secondary, we compared physiological responses and total training volume (measured as total completed repetitions) to add additional insight into the acute effects of the different protocols.

We hypothesized that the superset RT session would lead to a higher perceived effort and discomfort and be perceived less pleasurable and less enjoyable than the traditional RT session both midway and post-exercise. Additionally, we hypothesized that the superset RT session would reduce the training volume (i.e., reduce total number of repetitions in session), reduce total time to conduct the session, induce higher blood lactate levels and a higher average heart rate compared to the traditional RT session both midway and post-exercise. Lastly, and based on the affective responses, we expected that most participants would prefer the traditional RT session.

## 2 Materials and methods

### 2.1 Study design

The study used a randomized and counterbalanced, within-subject cross-over design. Participants were required to take part in a familiarization session followed by two experimental sessions (i.e., traditional and superset). The RT program consisted of eight exercises, targeting major muscle groups. The exercises were performed with two sets at approximately 10 repetition maximum (RM). The number of sets (2 sets) and the intensity (10-RM) was chosen as it is in the middle of the recommended range for untrained individuals to gain strength and hypertrophy (25). In the traditional session, the rest interval was two minutes between each set and exercise. In the superset session, two consecutive exercises were executed immediately after each other before a similar two minutes rest interval was given. Otherwise, the experimental sessions were identical.

**Figure 1:**
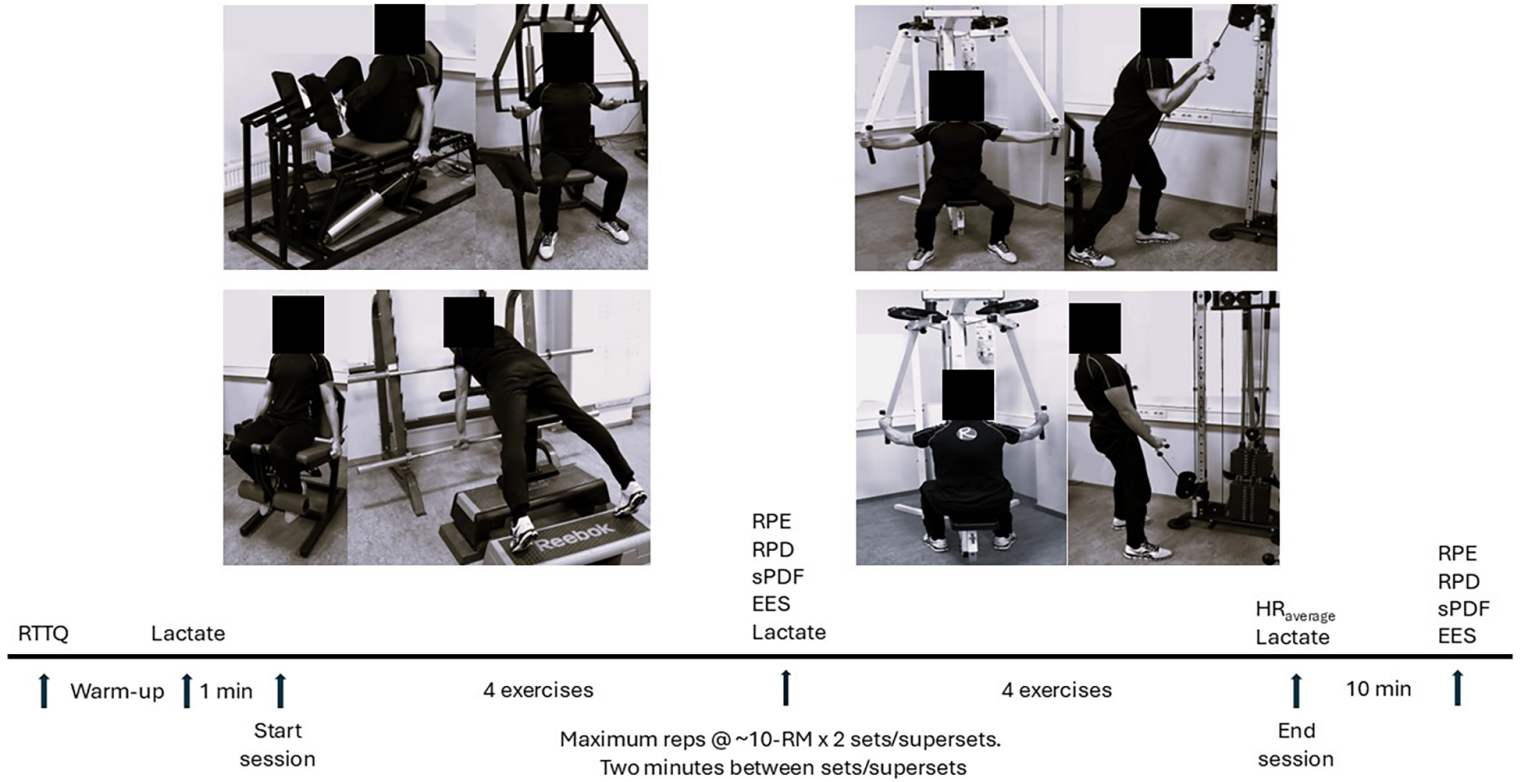
Schematic illustration of the design of the study.

In the experimental sessions the participants reported their perception of effort (RPE), discomfort (RPD), pleasure/displeasure (sPDF) and enjoyment (EES) in the middle and at the end of the sessions. Blood lactate was measured before, in the middle and at the end of each session, and the average heart rate was recorded for the entire sessions. Additionally, successful repetitions per set (training volume) and total training duration were measured (the training duration was rounded off to the nearest minute). The total numbers of completed repetitions per session was used as training volume since all other intra-exercise variables were held constant (26–28). See Figure 1 for an overview of the design.

### 2.2 Participants

30 untrained adults (17 females and 13 males) were recruited to participate in the study (Table 1). *A priori* power analysis estimated a requirement of 28 participants. The power analysis was performed in SPSS (IBM Corp. Released 2022. IBM SPSS Statistics for Windows, Version 29.0. Armonk, NY: IBM Corp.) and based on data material from previous study reporting a difference of 1.0 in RPD (traditional; 5.4 ± 2.2 vs. superset; 6.4 ± 2.0) with alpha level of 0.05, power of 0.8 and expected Pearson product-moment correlation (r) of 0.63 (19). To be included in the study participants had to be over 18 years old, be untrained (no regular training the last three months) and not have any injuries or pain that prohibited maximal exertion. Before being enrolled in the study, all participants were informed orally and in written form and provided a written consent. The study procedure was approved by the Norwegian Centre of Research Data (ref nr 322661) and was carried out in accordance with the University College's ethical guidelines.

**Table 1:** Anthropometrics (mean ± standard deviation).

	All (*n* = 30)	Males (*n* = 13)	Females (*n* = 17)
Age (years)	28.6 ± 7.0	31.0 ± 7.1	26.7 ± 6.5
Height (m)	1.72 ± 0.1	1.80 ± 0.8	1.66 ± 0.7
Body mass (kg)	77.4 ± 15.9	83.4 ± 12.7	72.9 ± 16.9
BMI	26.2 ± 5.2	25.8 ± 4.0	26.6 ± 6.0
Body fat (%)	27.3 ± 8.7	20.1 ± 5.6	32.2 ± 7.2

M, meters; kg, kilograms; BMI, body mass index.

### 2.3 Procedures

#### 2.3.1 Familiarization

In the familiarization session anthropometrics were measured and participants were made familiar with the questionnaires, scales, the procedures of the two experimental sessions and the physiological measurements. All exercises were familiarized with a progressive loading to estimate their ∼10-RM loads. For each exercise the participants started performing a set consisting of 10 repetitions at a low load to familiarize themself with the movement. After three minutes' pause the load was increased and the participant performed another set of 10 repetitions. This process continued until the participant and test leader agreed that the ∼10-RM was obtained. Normally this was achieved in 1–3 sets. The eight exercises were (in chronological order) (1) leg press, (2) chest press, (3) knee extension, (4) seal row, (5) flies, (6) cable triceps extension, (7) reversed flies and (8) cable biceps curl. In the superset session, exercise 1 and 2, 3 and 4, 5 and 6, 7 and 8 were performed as supersets. Furthermore, the exercises were performed using resistance training machines, except for the seal row where the participant lay on the stomach on an inclined bench and pulled a barbell upwards. For the RT exercises leg press and push-down, a 90°-degree angle of the knee/hip and elbow was used. Otherwise, the exercises were performed with a full range of motion. Individual standardizations were noted to keep the execution of repetitions within the set and between sessions as identical as possible. Importantly, the same load (10-RM) was used in both sessions and repetitions in each set were performed to failure.

#### 2.3.2 Experimental sessions

Participants were asked to refrain from alcohol and RT 48 h prior to each session and were encouraged to eat and sleep in the same manner before the sessions. As best as possible, the exercise sessions were conducted at the same time of the day. The participants were asked to report on the readiness to train questionnaire before each exercise session began to control for similar baseline levels. The questionnaire consisted of seven questions, which were formulated and anchored in the same manner as Pedersen et al. (29). Analysis detected no differences in the readiness to train between the two experimental sessions (*p* = 0.38–0.91).

The exercise sessions were performed within 4–15 days. The participants were instructed to contact the test leader if for any reason they were unable to participate on the arranged date (e.g., sickness, muscle soreness), and a new date was set approximately one week later. The standardized warm-up was accustomed from a similar study (19) and consisted of two sets each of the exercise's leg press, chest press and seal row with a 1-min rest interval between sets. First set were performed with 10 repetitions at 40% of 10-RM load and the second set with 10 repetitions at 60% of 10-RM load. After the last warm-up set, there was a 2-min rest interval before the first exercise started. Independent of RT session, the participants were instructed to perform repetitions continuously until voluntary failure in each set in a self-selected but controlled tempo (19). The test leader counted the repetitions, kept track of time, presented the scales and took the blood lactate measurements. To keep the experimental sessions as similar as possible, all sessions were conducted in a lab with only the participant and the same test leader present.

The participants were e-mailed 48 h after the last experimental session with the following question: “*If you had to choose one of the two training sessions as your regular training session, which would you prefer, and what is the main reason for this choice?*” The participants responded by replying to the mail. The answers were compiled and grouped together based on the theme of the explanation (19).

#### 2.3.3 Affective measurements

The questionnaires and scales were anchored and presented in the same manner as a prior study (19). The perceptive questionnaires were presented in the same way for all participants and in both sessions. The scales were presented in the following order: RPE, RPD, sPDF and EES. The participants were told to answer their subjective assessment of the specific affection in the middle and 10 min after the session. The test leader read the participants the questions and anchoring while presenting/showing the scales and question and anchoring at the same time. The RPE and RPD scales consist of a 11-point scale which were ranged from no effort/discomfort (0) to maximal effort/discomfort (10) (30). The RPE scale was presented with the following question: “*How much of your perceived physical capacity out of your perceived maximum (10 being your maximum) did you invest to complete this workout?*”. The scales upper and lower limit were anchored by the following phrase “*0 can be described as sitting still during the whole session while 10 would be maximal effort using your maximal physical capacity throughout the whole session*”. The RPD scale was presented with the following question: “*Based on the completed session, how much discomfort did you feel? The scale ends at 10 which could be described as you could not imagine the sensations relating to physical activity being any more intense?*” The scales upper and lower limit were anchored by the following phrase “*0 can be described as feeling no noticeable sensation relating to the training while 10 would be the most intense training related sensation you could imagine*”. The perceived session pleasure/displeasure (sPDF) was presented with the following question: “*How was your workout?*” (31). An 11-point scale stretching from −5 to +5; where a score of 0 is considered neutral, positive numbers (+1 to +5) represents pleasurable feelings and negative numbers (−1 to −5) represents unpleasurable feelings (32). The scales upper and lower limit were anchored by the following phrase “−*5 can be described as perceiving the session as one of the worst/least pleasurable training sessions you have ever conducted while 5 would be one of the best/most pleasurable training sessions you have ever conducted*”. Enjoyment was measured using the exercise enjoyment scale (EES), a seven-point scale ranging from 1 (“not at all”) – 7 (“extraordinarily”). The EES scale was presented with the following question “*How much did you enjoy the exercise session?*” (33). The scales upper and lower limit were anchored by the following phrase “*1 can be described as perceiving the session as one of the least enjoyable training sessions you have ever conducted while seven would be one of the most enjoyable training sessions you have ever conducted*”.

#### 2.3.4 Physiological measurements

Blood lactate was measured using Lactate Pro 2 (Arkray, Kyota, Japan) and corresponding lactate strips. Blood samples were collected from the fingertip of the participants. According to manufacturer's recommendation, the puncture site was cleaned with water and dried off with a paper towel. The first drop of the blood was wiped off with a new paper towel, while the measurement was taken on the second drop of blood (16). Measurements were taken after the warm-up (i.e., one minute before the exercise session started), immediately after the second set of the fourth exercise (i.e., middle of the session), and immediately after the last exercise (16, 22).

Heart rate was measured using Polar® M400 (Polar Electro Oy, Kempele, Finland) and corresponding Polar® H7 heart sensor chest band (1,000 Hz). The heart rate monitor was started immediately before the first set of the first exercise and stopped immediately after the last set of the last exercise. The average heart rate was used for further analysis.

### 2.4 Statistical analysis

All statistical analysis were performed using SPSS (IBM Corp. Released 2022. IBM SPSS Statistics for Windows, Version 29.0. Armonk, NY: IBM Corp.). Normality was assessed by visual inspection and Q-Q plot for the continuous variables (anthropometrics, repetitions, blood lactate, heart rate, time). Paired sample *t*-tests were used to assess possible differences in repetitions and heart rate between the two sessions. For the blood lactate, a 3 × 2 (time; before, middle and end of session × modality; traditional and superset) within subject, repeated analysis of variance (ANOVA) was used. When interactions or main effects were detected, Bonferroni *post hoc* corrections were applied. The continuous variables are presented as means ± standard deviations.

The ordinal data (RPE, RPD, sPDF, EES) are presented as median + interquartile range. For the affective measurements the Friedman's ANOVA was used to asses if differences existed within and between the sessions. If differences were detected, the Wilcoxon signed rank test was used for *post hoc* analysis and for the readiness to train questionnaire. To prevent type-1 error inflation, the Bonferroni-correction was applied for the alpha-level in *post hoc* analysis (i.e., multiplying the *p*-value by 4).

Effect sizes were calculated for all tests between sessions or test points (*t*-tests, Bonferroni *post hoc* corrections and Wilcoxon signed rank test) For the continues variables Cohen's d effect size (d) was calculated using the following equation: mean pre - mean post divided by the pooled standard deviations of the two. An effect size of 0.2–0.49 was considered small, 0.5–0.79 medium and ≥0.8 large (34). Effect size (r) for the ordinal data was calculated as the product-movement r with the following equation: r = z/√ n, with z being the *z*-value for the Wilcoxon signed ranked test and *n* being the number of participants. An effect size r of 0.1–0.29 was considered small, 0.3–0.49 medium and ≥0.5 large (34). Statistical significance was accepted at *p* < 0.05.

## 3 Results

**Figure 2:**
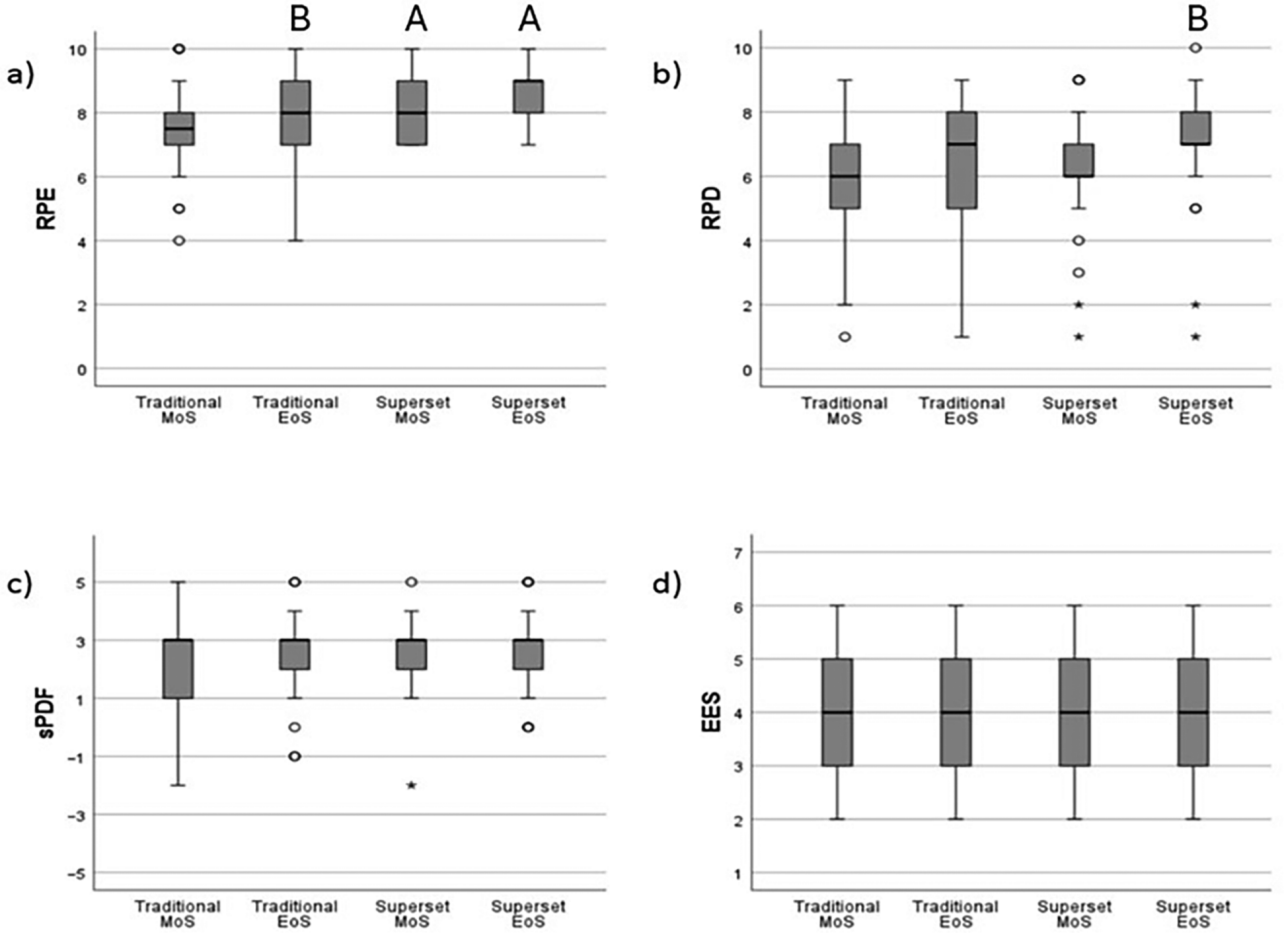
Affective responses to traditional and superset resistance training. **(a)** RPE, rating of perceived exertion, **(b)** RPD, rating of perceived discomfort, **(c)** sPDF, session pleasure/displeasure feeling scale, **(d)** EES, exercise enjoyment scale. A, significant difference from traditional session at same time point (*p* < 0.05), B, significant different from middle of session within same session (*p* < 0.05). MoS, middle of session, EoS, End of Session, ^o^Outlier higher than 1.5 multiplied by the interquartile range, *Extreme outlier higher than 3 multiplied by the interquartile range.

There was a statistically significant interaction effect in RPE (*p* < 0.001) and discomfort RPD (*p* < 0.001), while there were no differences for sPDF (*p* = 0.404) or EES (*p* = 0.829) within or between the two experimental sessions. All details are presented in Figure 2.

Comparing effort (RPE) between sessions, *post hoc* corrections demonstrated a statistically significant higher effort in the superset RT at the middle (*p* = 0.012, *r* = 0.54) and post-exercise (*p* = 0.016, *r* = 0.53) compared to traditional RT. For the traditional RT session, the effort increased significantly from the middle to post-exercise (*p* = 0.028, *r* = 0.49). No difference between middle and post-exercise were observed for the superset RT session (*p* = 0.368, *r* = 0.31).

For discomfort (RPD), no statistically significant difference was observed between the sessions in the middle (*p* = 0.336, *r* = 0.32) or at the end of the sessions (*p* = 0.092, *r* = 0.41). However, a statistically significant difference was demonstrated from the midway to post-exercise of the superset session (*p* < 0.001, *r* = 0.62), but not within the traditional session (*p* = 0.128, *r* = 0.39).

For blood lactate, there was an interaction effect between time and modality (F = 11.432, *p* < 0.001, Table 2). The *post hoc* analyses showed no difference between the modalities before the start of the sessions (*p* = 0.370, *d* = 0.17, Table 2). However, in the middle of the session (12.9%, *p* < 0.001, *d* = 0.73) and at the end of the sessions (18.3% *p* < 0.001, *d* = 0.95), the superset session led to higher lactate values than the traditional session. When analyzing the lactate levels within each modality we observed the same pattern for both modalities. Both sessions led to higher lactate levels at middle (Traditional; 262.5%, *p* < 0.001, *d* = 2.56, Superset; 296.6%, *p* < 0.001, *d* = 3.47) and end of the session (Traditional; 240.6% *p* < 0.001, *d* = 2.59, Superset; 290.9% *p* < 0.001, *d* = 3.74), compared to before the start of the session. There were no differences between the middle and the end of session for any of the modalities (Traditional; *p* = 0.265, *d* = 0.32, Superset; *p* = 1.000, *d* = 0.12).

**Table 2:** Blood lactate values (in mmol/L) for the experimental protocols (mean ± standard deviation).

	Start of sessions	Middle of sessions	End of sessions	Change start to end
Traditional	3.2 ± 1.1	11.6 ± 4.0*	10.9 ± 3.7*	7.7 ± 3.0
Superset	3.3 ± 1.0	13.1 ± 3.5*,**	12.9 ± 3.1*,**	9.6 ± 2.6

* Significantly different from pre-exercise within same modality (*p* < 0.05).

** Significantly different from traditional (*p* < 0.05).

The traditional session had a mean heart rate of 128 ± 16 beat per minute (bpm), while the superset session had a mean heart rate of 138 ± 16 bpm. The mean heart rate in the superset session was significantly higher compared to the traditional session (9.9 ± 7.2 bpm, *p* < 0.001, *d* = 1.53).

Comparing the total repetitions between sessions, the traditional session had 4.6% more repetitions compared to the superset session (158 ± 18 vs. 151 ± 17 repetitions, *p* = 0.006, *d* = 0.54, Table 3), and took 60% longer time to complete (40 ± 2 vs. 25 ± 2 min, *p* < 0.001, *d* = 6.62).

**Table 3:** Average repetitions per set performed with ∼10-RM loading (mean ± standard deviation).

	Leg press	Seated bench press	Leg extension	Seal row	Flies	Cable triceps extension	Reversed flies	Cable biceps curl
Traditional	13.4 ± 4.7	8.7 ± 2.6	10.6 ± 1.9	9.6 ± 1.8	9.0 ± 2.7	10.3 ± 2.7	8.7 ± 2.0	8.8 ± 2.6
Superset	12.4 ± 4.6	8.8 ± 1.9	10.8 ± 2.7	9.5 ± 2.1	8.4 ± 2.6	9.2 ± 2.5	8 ± 1.9	7.9 ± 1.8

Finally, twenty out of the thirty participants reported that they would prefer to continue with superset training if having to choose between the two.

## 4 Discussion

Consistent with our hypothesis and other studies (14–16, 19), the perceived effort in the superset session in the present study was higher compared to the traditional session. The higher perceived effort in the superset session could be explained by the more intense workout, i.e., same work in a shorter period of time. This would increase the metabolic stress which has been coupled with increased rating of perceived effort (16). This speculation corresponds with our observations of increased levels of blood lactate and average heart rate for the session. Higher levels of blood lactate have been associated with higher perception of effort (15, 16), while heart rate has shown strong relationship with the perception of effort in multiple sports (35). These higher levels of metabolic stress could also explain the tendency for the increased perception of discomfort for the superset session. Higher levels of metabolic stress have shown to increase the perception of fatigue and pain/discomfort (36). Furthermore, it is reported that the perceived effort and discomfort have a significant, but weak correlation to each other (30). Therefore, the increase in both could partly be explained by each other.

Notably, the increased perception of effort and discomfort did not lead to a change in perception of pleasure/displeasure or enjoyment between the sessions. These findings were in contrasts with previous studies indicating that the perception of effort has a negative association with the feelings of pleasure/displeasure scale (32, 37, 38). For example, Almeida et al. (38) compared traditional resistance training with more intense modalities using shorter rest intervals (rest-pause training) or shorter rest intervals and reducing intensity (sarcoplasmatic stimulating training) in male bodybuilders. The results showed an increased perception of effort and discomfort, and reduced pleasure for the two more intense modalities compared to traditional training. Importantly, the differences in study design and training experience makes it difficult to compare the findings to our results. More similar to our design, Andersen et al. (19) compared the perception of pleasure/displeasure (sPDF) and enjoyment (EES) between superset and traditional RT in a whole-body RT session and demonstrated a tendency for the superset session to be perceived as more pleasurable (*p* = 0.059) while there was no change for enjoyment (*p* = 0.661). Of note, in contrast to our population, the population in Andersen et al. (19) had a mean average resistance training experience of 8.4 (±6.6) years, which may indicate they are more used to and find more pleasure in the more intense superset RT.

There were no changes in the perception of pleasure/displeasure or enjoyment from the middle to post-sessions. In contrast, most people (>95%) experience an immediate positive response (i.e., affective rebound effect) after cessation of aerobic exercise (39). It has been suggested that the affective rebound effect becomes evident after exercise leading to an affective decline. In our study the rating of perceived enjoyment and pleasure were generally high during the sessions, which arguably could reduce the potential of a rebound effect. These explanations are consistent with previous studies suggesting that the affective rebound effect is less pronounced in resistance training exercises (40, 41).

In the present study, a 4.4% decrease in total completed repetitions were observed in the superset session compared to traditional session when performing sets to voluntary failure. This is a similar decrease in repetitions (4.2%) as the participants in Andersen et al. (19). Of note, previous studies have reported similar volume (15, 42) or higher volume (13, 20) conducting superset compared to traditional RT. However, these studies are limited by only examining two exercises (13, 15, 20, 42). The findings in our study supports the notion that the decrease in volume first becomes apparent in RT sessions when including several exercises. This is strengthened when comparing the repetitions between the first two exercises in our study, resulting in no difference between the protocols (difference: 1.8 repetitions, *p* = 0.119).

The reduction of completed repetitions, i.e., training volume, found in our study could be explained by the increased metabolic stress (i.e., increased blood lactate and heart rate) as a result of reduced time to rest. Less time to rest would reduce the time to restitute between the sets/exercises and increase the metabolic and neuromuscular stress (43, 44). An increase in metabolic stress has been associated with a lower performance in volume due to reduced capacity to sustain muscular force (17). Our finding are comparable to several studies reporting an increased metabolic stress in superset compared to traditional RT (15, 16, 45). Although speculative, over findings may also be a result of increased central fatigue during the superset session (44).

The present study has several limitations which needs to be addressed. First, the participants in the study were unfamiliar with the scales and RT before the start of this project. The lack of RT experience may have affected their responses on the affective outcomes due to pre-assumed expectation, lack of sensation, or stable base-line references of the two RT sessions. Importantly, all participants were familiarized with the questions and scales before the experimental sessions. Furthermore, the sessions were randomized, and the comparisons were within participants possibly nulling out any familiarization effect. Secondly, the study aimed to target approximately 10-RM in each exercise. However, some of the participants in the experimental session managed to lift more repetitions (e.g., 13 and not 10 repetitions on average in the first exercise). We would preferred to have a more similar number of repetitions between the exercises, however and importantly, the sets in both modalities were conducted with the same loading to failure, and the order of the sessions were randomized (i.e., a systematic error affecting both RT sessions). Also, even if the number of repetitions/intensity to some degree extended 10-RM, it could still be argued to be well within an effective intensity for increasing muscle strength, as long as the sets were performed to failure (46). Furthermore, the short-term learning effects between familiarization- and the experimental sessions was potentially reduced by using training machines and not free-weights (47). Still, future studies should investigate individuals’ perceptions to superset across different loading and sets in addition to examine the impact on affective responses over time conducting the two RT session among untrained participants. Lastly, the participants in this study were untrained adults and the findings can therefore not be generalized to other populations.

Interestingly, 20 out of 30 participants choose superset as their preferred sessions if they were to continue RT. The main reasons given for this choice was time-efficiency and a feeling of a harder/more effectful workout. Among the 10 participants preferring traditional RT, the main reasons were a feeling of being able to perform better with the traditional RT and the superset RT was perceived as too strenuous to continue over time. Considering that superset leads to greater metabolic stress (i.e., blood lactate and heart rate) and higher perceived effort, it is of interest how untrained individuals would adhere to superset RT over time. Therefore, from a public health perspective, it would be of interest to conduct longitudinal studies to investigate the efficacy and adherence of prescribing superset as RT modality to untrained adults in real-world settings (i.e., without supervision sessions).

In conclusion, one single session of superset RT was perceived as more effortful and led to higher levels of blood lactate, increased average heart rate, a decrease in volume and took less time compared to a single session of traditional RT in untrained adults. There were no statistically significant differences between the sessions for discomfort, session pleasure/displeasure or enjoyment, however, 20 out of the 30 participants preferred the superset session.

## Data Availability

The raw data supporting the conclusions of this article will be made available by the authors, without undue reservation.
